# Blockchain-Enabled Identity Based Authentication Scheme for Cellular Connected Drones

**DOI:** 10.3390/s25226935

**Published:** 2025-11-13

**Authors:** Yu Su, Zeyuan Li, Yufei Zhang, Xun Gui, Xue Deng, Jun Fu

**Affiliations:** 1 China Mobile Chengdu Institute of Research and Development, Chengdu 610041, China; suyu@cmii.chinamobile.com (Y.S.); zhangyufei@cmii.chinamobile.com (Y.Z.); fujun@cmii.chinamobile.com (J.F.); 2 School of Cyber Security, University of Electronic Science and Technology of China, Chengdu 611731, China; guinh3@uestc.edu.cn (X.G.); dengxue1105@163.com (X.D.)

**Keywords:** cellular connected drones, IBC, blockchain, MQTT

## Abstract

The proliferation of drones across precision agriculture, disaster response operations, and delivery services has accentuated the critical need for secure communication frameworks. Due to the limited computational capabilities of drones and the fragility of real-time wireless communication networks, the cellular connected drones confront mounting cybersecurity threats. Traditional authentication mechanisms, such as public-key infrastructure-based authentication, and identity-based authentication, are centralized and have high computational costs, which may result in single point of failure. To address these issues, this paper proposes a blockchain-enabled authentication and key agreement scheme for cellular-connected drones. Leveraging identity-based cryptography (IBC) and the Message Queuing Telemetry Transport (MQTT), the scheme flow is optimized to reduce the communication rounds in the authentication. By integrating MQTT brokers with the blockchain, it enables drones to authenticate through any network node, thereby enhancing system scalability and availability. Additionally, cryptographic performance is optimized via precompiled smart contracts, enabling efficient execution of complex operations. Comprehensive experimental evaluations validate the performance, scalability, robustness, and resource efficiency of the proposed scheme, and show that the system delivers near-linear scalability and accelerated on-chain verification.

## 1. Introduction

The drones, also commonly known as unmanned aerial vehicles (UAVs), provides a wide range of services for drone applications, including delivery services, aerial surveys [1], traffic and environment monitoring, UAV-assisted wireless communication [2], air-to-ground wireless network coverage [3], as well as search and rescue operations. In 5G networks, drones for various applications are connected into the cellular networks as new aerial users, so that drones can be operated beyond line of sight [4]. To support these real-time services effectively, significant recent contributions have focused on optimizing the operational performance of these aerial networks. For UAV-assisted Wireless Powered Communication Networks (WPCNs), recent research has introduced sophisticated co-design strategies. These include a Hybrid TDMA/NOMA protocol for sensor transmission and a Clustering-based Dynamic Adjustment of the Shortest Path algorithm to jointly optimize power allocation, clustering of ground nodes, and UAV trajectory under limited battery capacity [5]. Alongside these crucial performance optimization efforts, data of drones are facing serious security challenges. The data may contain highly sensitive information, and the fragile communication network among drones makes the transmitted data easily intercepted and captured. The work in [6] surveys security challenges like DoS and Man-in-the-Middle attacks in drone communication and countermeasures using emerging technologies such as blockchain. The work in [7] examines secure communication protocols and challenges in UAV networks integrated with Internet of Things (IoT) environments. The work in [8] reviews security and privacy vulnerabilities specific to UAV systems, including threats to data integrity and confidentiality. To maintain secure communication in cellular connected drones, authentication and key agreement schemes are usually adopted, which provide a mechanism for communicating entities to have mutual authentication before other access control policies are implemented, thus fundamentally guaranteeing system security. The existing authentication schemes mainly include Public Key Infrastructure (PKI) [9,10], Identity-Based Cryptography (IBC) [11,12], and certificateless authentication [13].

Since PKI involves multiple stages such as certificate issuance, storage, maintenance, and authentication, it increases the total construction cost of the system. Furthermore, due to the highly dynamic and frequently changing network topology of the drone swarm, communication links are prone to frequent disconnection and reconnection. The bidirectional authentication process of PKI requires bidirectional transmission of certificate files, which not only reduces authentication efficiency but also further increases the system’s communication and computation cost. Lastly, PKI adopts a centralized system architecture, which not only severely limits system scalability but also high-lights single-point security bottlenecks in the network system.

IBC provides an identity-based encryption solution that reduces management overhead by directly generating public keys through the Key Generation Center (KGC), simplifying the certificate exchange and verification process. However, in resource-constrained drones with high real-time requirements, the authentication and key agreement processes still require multiple exchanges of computational parameters (drone ID, public key, secret parameters) and computations, resulting in low communication efficiency and high computational consumption, necessitating further optimization. Additionally, existing IBC-based drone identity authentication and key agreement systems are centralized, which not only severely limits system scalability but also facing the rise of single point of failure in the network system.

The certificateless authentication scheme represents an enhancement over identity-based public key cryptography. In the context of Mobile Ad-hoc Networks (MANET), studies such as [14,15,16,17] have integrated threshold cryptography with certificateless public key cryptography to develop authentication models. Nevertheless, the security of the system’s master key is dependent on the complete trustworthiness and integrity of the distributed server. Moreover, key negotiation processes in these frameworks are susceptible to man-in-the-middle attacks. Many of the approaches discussed in the aforementioned literature employ bilinear pairing, which offers strong security guarantees. Key distribution in such systems generally necessitates the use of a secure channel.

To address the issues, this paper first designs an authenticated key agreement protocol for cellular connected drones based on the Identity-based authentication scheme, which significantly reduces the number of communication rounds and computational operations during the authentication process, thereby enhancing the efficiency of identity authentication and key agreement. Leveraging the characteristics of blockchain such as decentralization, data immutability, and smart contracts, this paper constructs a blockchain-enabled distributed system for cellular connected drones authentication. This eliminates the single point of failure in existing drone identity authentication and key agreement systems based on PKI or IBC. Furthermore, this paper combines Message Queuing Telemetry Transport (MQTT) Broker cluster with blockchain system, decoupling the communication between drones and blockchain nodes. This enables drones to perform identity authentication, key agreement, and encrypted communication through any blockchain node, significantly enhancing the system’s availability and flexibility.

### 1.1. Contribution

The contributions of this paper are as follows:

1. An IBC-based authentication and key agreement scheme. We propose a novel authentication and key agreement protocol founded on IBC, specifically leveraging the standardized SM9 algorithm [18]. By optimizing the protocol flow, our scheme materially reduces the communication rounds and computational overhead inherent in the authentication process. This establishes a highly efficient and provably secure mechanism tailored for the operational constraints of resource-limited drone systems.
2. A decoupled and scalable system architecture. This paper introduces an innovative application-layer architecture that synergistically integrates an MQTT Broker cluster with the blockchain. Through a clustered publish/subscribe mechanism, this design effectively decouples the direct communication between drone clients and the blockchain access nodes. Consequently, it enables any drone to complete identity authentication, key agreement, and subsequent secure communications through any available network node, thereby enhancing the system’s high availability and horizontal scalability.
3. Performance optimization via precompiled smart contracts. To overcome the inherent performance limitations of executing complex cryptography within a standard smart contract virtual machine, we integrate the computationally demanding bilinear pairing algorithm into the blockchain’s core as a precompiled smart contract. Building upon this optimized cryptographic primitive, we designed a comprehensive suite of smart contracts to manage the entire identity lifecycle, encompassing drone registration, mutual authentication, and key updates. This method constructs a viable, high-performance, blockchain-enabled system for robust drone authentication and key management.

### 1.2. Organization of Paper

The remaining content of this paper is organized as follows. The recent related research on authentication for cellular connected drones is discussed in Section 2. Section 3 introduces the blockchain enabled drone authentication system model. Section 4 elaborates the drone identity authentication and key agreement scheme. Section 5 designs smart contracts for drone registration, identity authentication and key updates. Section 6 shows the security analysis of the proposed scheme. Furthermore, the proposed scheme is simulated in Section 7. Finally, the article is concluded in Section 8.

## 2. Related Works

Communication security serves as the core prerequisite for ensuring both mission execution and data safety in UAV systems. To address prevalent security threats such as jamming and eavesdropping, constructing an efficient and robust Authenticated Key Agreement (AKA) scheme has become a consensus in both academic and industrial communities. In recent research advancements, existing AKA schemes can be categorized into two major paradigms based on their trust models: the first follows the traditional cybersecurity approach, represented by PKI and IBC in centralized authentication schemes; the second consists of decentralized authentication schemes that have emerged to adapt to the distributed and dynamic nature of UAV networks, primarily exemplified by applications of blockchain technology. This section will provide an in-depth analysis of representative works from these two categories, systematically review their technical contributions and inherent limitations, thereby accurately identifying gaps in current research and clarifying the distinctive contributions of this work.

Traditional centralized schemes are widely adopted in the early stages of UAV security research. PKI relies on one or more centralized Certificate Authorities (CAs) to issue and manage digital certificates. For instance, Ozmen and Yavuz [15] contribute an optimized PKI framework for resource-constrained small UAVs, reducing energy consumption by up to 35% through precomputation and specific elliptic curves. However, PKI’s strong dependence on centralized CAs exposes fundamental architectural weaknesses in large-scale, highly dynamic scenarios such as UAV swarms. To mitigate these issues, subsequent studies proposed a series of improvements. For example, Verma et al. [16] introduce a short-certificate-based proxy signature (CB-PS) scheme aiming to enhance efficiency by reducing certificate size, while Das et al. [10], after analyzing serious security design flaws in prior schemes, propose an improved certificate-based universal access control scheme. Although these works optimized performance and security to some extent, they essentially remain partial fixes to the existing framework and fail to resolve the inherent architectural deficiencies rooted in centralized trust.

To circumvent the complex certificate management inherent in PKI, some research has shifted towards IBC, pioneered by Shamir [19], which allows the direct use of user identification as a public key. Wani et al. [20] apply this concept to UAV heterogeneous networks (HetNets), with their contribution being the design of an identity-based authentication mechanism to counter potential intruder threats. However, IBC schemes do not eliminate centralized dependence; instead, they shift the root of trust to a centralized KGC, which generates and holds the private keys for all entities in the network. This not only introduces a significant key escrow problem but also turns the KGC itself into a new high-value attack target and a single point of failure.

Given the inherent architectural limitations of centralized solutions, research has gradually shifted focus towards decentralized model paradigms. Among these, blockchain technology, leveraging its distributed, tamper-resistant, and traceable characteristics, has shown significant potential in the field of UAV authentication. Researchers have explored blockchain applications from multiple dimensions. Regarding fundamental authentication services, Wang et al. [21] proposed a Blockchain and Group Decentralized Identifiers assisted authentication scheme (BGAS), which significantly enhances the scalability of the authentication process and resistance to single points of failure; while Tan, Wang et al. [22] designed a blockchain-assisted distributed lightweight authentication service. Its core contribution lies in utilizing blockchain for the distributed and immutable storage of authentication information, and employing smart contracts to provide UAVs with convenient access and update operations for this information. To further address the potential performance overhead associated with on-chain interactions, Tan, Liu, and Kato [23], in subsequent work, propose a scheme incorporating blockchain edge nodes, where UAVs merely act as clients invoking APIs. The contribution of this work is the significant reduction of resource consumption on the UAV side. In addressing node load balancing, Kandi et al. [24], propose a novel decentralized blockchain-based key management protocol for IoT, which balances the load among nodes according to their capabilities. Concerning specific functionalities and architectural enhancements, Li et al. [25] tackle the challenge of group key distribution over unreliable channels, contributing a mutual-healing group key distribution scheme based on a private chain. For cross-domain authentication, the work by Xie et al. [26] focuses on the lightweight cross-domain authentication problem in multi-UAV wireless networks, proposing a dual blockchain-assisted trusted authentication scheme; while Du et al. [27] proposed a fog node-assisted blockchain authentication scheme, where edge fog nodes act as proxies for UAVs to perform registration and authentication on the chain, enabling seamless authentication across regions. Furthermore, Shahidinejad and Abawajy [28] addresse the issue that many existing schemes lose critical privacy attributes, such as anonymity, when integrating blockchain. They contributed a cost-effective anonymous authentication protocol by adopting Chebyshev chaotic maps as the cryptographic system.

While the application of blockchain has brought substantial advancements across various layers, the concomitant issues, such as increased burden on UAVs, and heightened dependence on other nodes and the GSS network, cannot be overlooked.

Through a systematic review of the aforementioned works, we identify that current UAV authentication schemes still exhibit significant limitations in various aspects, as summarized in Table 1. While PKI and IBC schemes suffer from the bottleneck of centralization, the listed blockchain-based solutions achieve decentralized authentication but still face several challenges. These include latency in on-chain transactions, throughput bottlenecks in smart contract execution, and the tight coupling between the authentication process and blockchain interactions. Particularly noteworthy is the disconnection between existing authentication frameworks and application-layer communication protocols. In typical scenarios such as real-time telemetry and command-and-control for UAVs, event-driven IoT messaging protocols like MQTT are widely adopted. However, current security schemes fail to provide deeply integrated, native authentication and key agreement mechanisms tailored for them. As a result, achieving secure and efficient communication authentication remains an unresolved challenge within cellular-IoT converged networks.

To fill this gap, this paper innovatively proposes an integrated blockchain and MQTT-based authentication and key agreement scheme for cellular-connected UAVs. In contrast to existing works, the differential contribution of our scheme lies in its novel co-design framework. While leveraging blockchain technology for decentralized authentication, it natively integrates the authentication and key agreement process with the MQTT messaging protocol. This achieves decoupling between communication and consensus, thereby enabling efficient, asynchronous, and scalable event-driven communication in large-scale, highly dynamic cellular network environments. The proposed approach of deeply integrating a decentralized trust root with a scalable communication paradigm represents a direction that has not been sufficiently explored in previous works.

## 3. System Model

**Figure 1:**
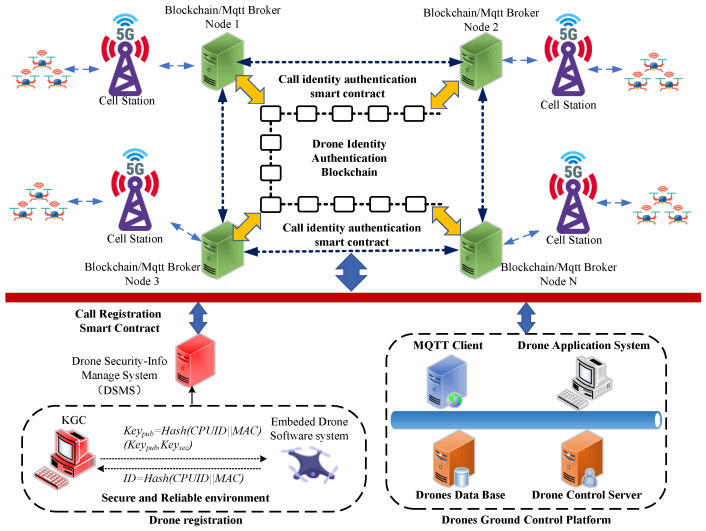
Proposed System Model.

In this section, we introduce the structure of the proposed authentication scheme. As shown in Figure 1, this paper designs an authentication system that integrates blockchain node clusters and MQTT broker node clusters.

In the drone registration stage, in a secure and reliable environment, as shown in the bottom side of Figure 1, the drone’s onboard terminal obtains its own MAC address and CPUID information through its internal embedded software system. Subsequently, it generates a unique ID=Hash(CPUID||MAC) for the drone, and transmits this ID information securely to the KGC. The KGC then generates the drone’s public-private key pair $\left(Key_{pub},Key_{sec}\right)$ from the received ID information and securely transmits it back to the drone terminal for confidential storage. This process is typically done offline. Meanwhile, the drone’s public-private key pair information $\left(Key_{pub},Key_{sec}\right)$ is uploaded to the Drone Security Information Management System (DSMS). The DSMS then writes the drone information into the blockchain system by triggering a system registration smart contract.

In the blockchain system, to ensure the registered drones can perform identity authentication, key agreement, and encrypted communication through any blockchain node, each blockchian node is designed with an MQTT broker service. All MQTT brokers of the blockchain nodes form a Broker cluster. Thus, the drones ground control platform which provides various applications of drones, can communicate with drone connected with cellular network accessing any node of blockchain, after passing identity authentication and key agreement on the MQTT broker.

## 4. Authentication and Key Agreement

In this section, we introduce the proposed authentication and key agreement scheme in detail.

### 4.1. System Initialization

**Figure 2:**
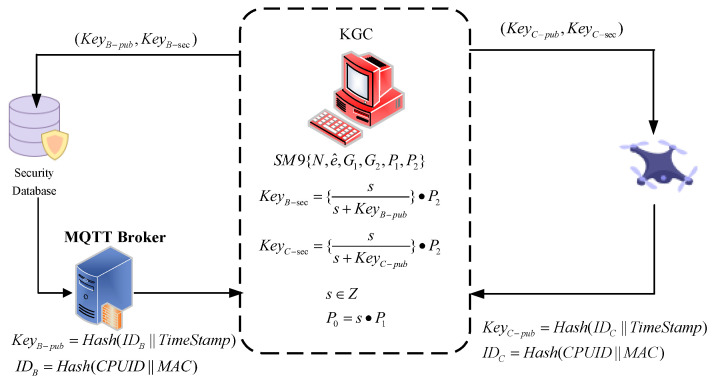
Initialization of the Proposed System.

The initialization stage of the system involves acquiring the unique ID information of the client (drone) and MQTT service terminal, generating public key and private key from the KGC. The process is outlined in Figure 2.

Step 1: Initialization of Elliptic Curve Information: KGC, the drone, and the MQTT broker initialize the elliptic curve information using the same parameters. This includes adopting the 256-bit BN curve recommended by the ISO18033-5 AMENDMENT 1 standard [18], selecting two additive cyclic groups G1 and G2 of order *N* with generators P1 and P2, and choosing a bilinear map *e* that satisfies e:G1×G2→G.

Step 2: Generation of Encryption Keys: KGC generates the encryption master private key *s*, a large random prime number, using a secure random number generator. The encryption master public key *P* is then calculated as $P_{0}=s\cdot P_{1}$. With the encryption master private key, KGC generates key pairs as follows.

(1) $$Key_{pub}=\mathrm{Hash}\left(ID||TimeStamp\right)$$

(2) $$Key_{sec}=\frac{s}{s+key_{pub}}\cdot P_{2}$$

Step 3: Unique ID Generation and Key Pair Allocation: The client (drone) and MQTT broker employ their CPU serial number and MAC address to generate a 32-byte unique ID using a hash function: ID=Hash(CPUID||MAC). This ID uniquely identifies each UAV in the system. The IDs are then sent to KGC, which generates separate public-private key pairs $\left(Key_{C-pub},Key_{C-sec}\right)$ for the client (drone) and $\left(Key_{B-pub},Key_{B-sec}\right)$ for the MQTT Broker. The inclusion of a timestamp in the key generation process ensures that each key pair is unique.

Step 4: Key Pair Retrieval: The client and MQTT broker retrieve their respective key pairs using their IDs (which are also their public keys). This is typically done by accessing a database or securely copying files from a secure environment. The retrieved key pairs are then stored securely and used for secure communication between the client and the MQTT broker.

### 4.2. Authentication

**Figure 3:**
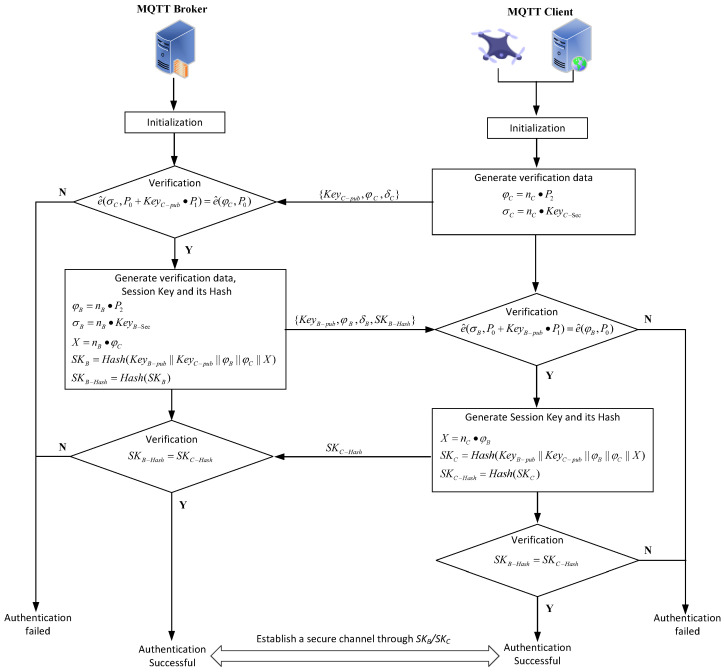
Authentication Flowchat of the Proposed System.

The process of identity authentication is depicted in Figure 3 and outlined as follows.

Step 1: The client (drone) generates a random number $n_{C}$ and authentication information $\left\{\phi_{C},\sigma_{C}\right\}$, where $\phi_{C}=n_{C}\cdot P_{2}$, $\sigma_{C}=n_{C}\cdot Key_{C-sec}$. $\left\{Key_{C-pub},\phi_{C},\sigma_{C}\right\}$ is sent to the server for authentication.

Step 2: Upon receiving the client’s authentication information, the GCS (MQTT Broker) performs a bilinear pairing check $\hat{e}\left(\sigma_{C},P_{0}+Key_{C-pub}\cdot P_{1}\right)=\hat{e}\left(\phi_{C},P_{0}\right)$. If the check is successful, the server generates a random number $n_{B}$ and authentication messages $\phi_{B},\sigma_{B}$, where $\phi_{B}=n_{B}\cdot P_{2}$ and $\sigma_{B}=n_{B}\cdot Key_{B-sec}$. Additionally, a session key and its hash value can be represented as $X=n_{B}\cdot \phi_{B}$, $sk_{B}=\mathrm{Hash}(Key_{B-pub}||Key_{C-pub}\left|\right|\phi_{B}\left|\right|\phi_{C}\left||X\right)$, $sk_{B-Hash}=Hash\left(sk_{B}\right)$. The server then sends the authentication messages and the session key hash value $\left\{Key_{B-pub},\phi_{B},\sigma_{B},sk_{B-hash}\right\}$ to the client. If the authentication fails, the process terminates.

Step 3: The client, upon receiving the server’s authentication messages, performs a similar bilinear pairing check, $\hat{e}\left(\sigma_{B},P_{0}+Key_{B-pub}\cdot P_{1}\right)=\hat{e}\left(\phi_{B},P_{0}\right)$. If successful, it generates a session key and its hash value $X=n_{C}\cdot \phi_{B}$, $sk_{C}=\mathrm{Hash}(Key_{B-pub}||Key_{C-pub}\left|\right|\phi_{B}\left|\right|\phi_{C}\left||X\right)$, $sk_{C-Hash}=Hash\left(sk_{C}\right)$. If $sk_{B-\mathrm{Hash}}=sk_{C-\mathrm{Hash}}$, the identity authentication is deemed successful, and the client sends the session key hash value to the server.

Step 4: The server verifies the received $sk_{C-\mathrm{Hash}}$ against its own $sk_{B-\mathrm{Hash}}$. If they match, the identity authentication is successful, and $sk_{B}$ and $sk_{C}$ are established as the symmetric keys for secure communication between the client and the server.

### 4.3. Session Topic Secret Key Derivation

**Figure 4:**
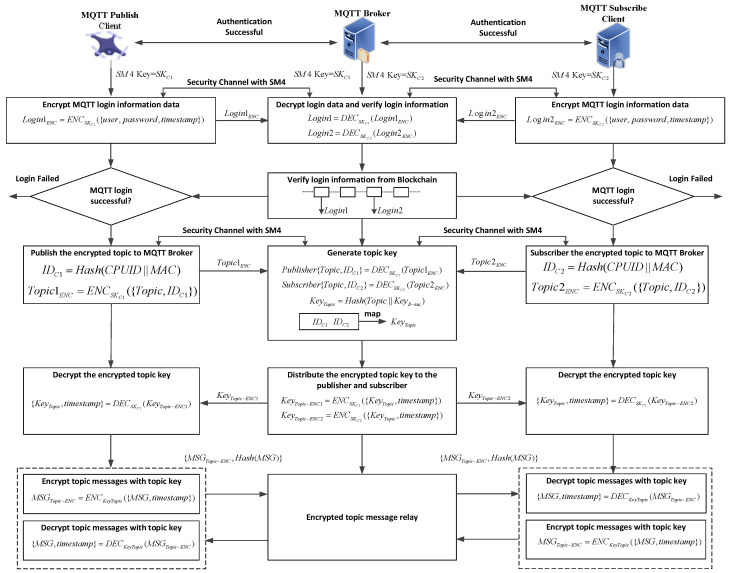
Session Topic Secret Key Derivation and Data Communication.

The process of the session topic secret key derivation and the communication data encryption/decryption transmission is illustrated in Figure 4. The clients in Figure 4 are the MQTT Publish Client (drone), the MQTT Subscribe Client (GCS app or drone), and the MQTT Broker (GCS server).

Step 1: When the client and server identity authentication is successful, corresponding symmetric session keys Sk1 and Sk2 are generated. During the MQTT authentication phase, the clients encrypt their respective usernames, passwords, and the current timestamp using symmetric key algorithm, generating login encryption packets Login1ENC and Login2 ENC. These are then placed in the CONNECT data packet and sent to the MQTT server.

Step 2: Upon receiving the CONNECT data packet, the server decrypts the login information using the corresponding session keys of the clients, retrieves the {user,password,timestamp} of the respective clients, and checks the timestamp. If the timestamp is within the specified range, the login data is considered valid, and the server proceeds with the MQTT login using the decrypted user and password. Upon successful login, the server returns a CONNACK message to the client. If the timestamp is outside the specified range, the login data packet is considered expired, and the MQTT server returns a login failure. This timestamp verification effectively prevents replay attacks.

Step 3: After successful MQTT login, the MQTT topic secret key is generated. In this stage, the client sends the device ID and the publish/subscribe topic (Topic) to the server. The client encrypts the ID and Topic information using the session key: $Topic1_{ENC}=ENC_{SK-C1}\left(Topic,ID_{C1}\right)$, $Topic2_{ENC}=ENC_{SK-C2}\left(Topic,ID_{C2}\right)$, and sends it to the server. The server decrypts the information using the client’s different session keys, and generates the topic session secret key using its own private key $Key_{B-sec}$ and the Topic: $Key_{Topic}=\mathrm{Hash}(Topic||Key_{B-sec})$. Then, the server constructs a topic information mapping using the client’s ID information (ID1 and ID2).

Step 4: The server encrypts the topic secret key KeyTopic and timestamp using the session secret key and sends it to the client. The client decrypts the information using the session key to obtain the topic secret key KeyTopic. This completes the topic secret key derivation and distribution stage.

### 4.4. Communication Data Encryption and Decryption Transmission

This stage involves encrypting and decrypting the main message with the topic key, realizing the entire MQTT publish-subscribe process.

Step 1: The subscriber sends a data packet to the server to subscribe to the specified topic data.

Step 2: The publisher packages the corresponding information MSG and timestamp, {MSG,timeStamp}, and uses the $Key_{Topic}$ of the corresponding topic as the SM4 encryption key to encrypt data packet: $MSGtopic_{ENC}=ENC_{KeyTopic}\left(\left\{MSG,timeStamp\right\}\right)$, and then sends the published information back to the server.

Step 3: Once the server receives the data packet published by the publisher, it forwards the data packet to the subscriber of the corresponding topic through an internal mapping. The subscriber decrypts the received $MSGtopic_{ENC}$ data packet using KeyTopic to obtain MSG and timeStamp. After checking that the timestamp is within the specified range, the information is adopted, thus completing the entire MQTT publish-subscribe process.

### 4.5. Key Refreshment

Despite the relatively short lifespan of drone networks, the risk of key compromise due to prolonged use of the same key cannot be overlooked, particularly in the open environment of wireless networks, where side-channel attacks and other attempts to decrypt the key are prevalent. Therefore, it is crucial to establish a fixed time interval for updating the key pairs of nodes once the specified duration has elapsed.

Taking the example of a key refresh request initiated by a ground station to drone node C, the steps involved in the key refresh stage are outlined below:

Step 1: KGC utilizes the unique ID and TimeStamp to regenerate a certification key pair. The new key pair $\left\{Key_{pub},Key_{sec},ID\right\}$ is then transmitted to drone via an established secure channel.

Step 2: Upon receiving the new key pair $\left\{Key_{pub},Key_{sec},ID\right\}$ through an encrypted channel, drone node C decrypts it and performs a bilinear pairing calculation to verify if the key was indeed generated by KGC. Subsequently, it checks if the ID matches the hash value Hash(CPUID||MAC) derived from its own CPUID and MAC address. If the IDs match, the drone node updates and stores the certification key pair.

## 5. Key Management on Blockchain

In this section, the implementation of DSMS is introduced.

### 5.1. Blockchain Node Module Struture

FISCO BCOS (Financial Blockchain Shenzhen Consortium Blockchain Open Source) is an enterprise-level blockchain platform developed by China Financial Blockchain Consortium [29]. It is designed to provide high performance, high security, and high scalability solutions for financial institutions and other industries. The platform achieves up to thousands of Transactions Per Second (TPS) through optimized consensus algorithms and efficient network communication mechanisms. FISCO BCOS supports multiple encryption algorithms and security protocols, combined with multi-layered permission management and auditing mechanisms to ensure data security. As a highly customizable and secure blockchain platform, FISCO BCOS can be applied to solve data security problems faced by industry, energy, finance and other fields.

**Figure 5:**
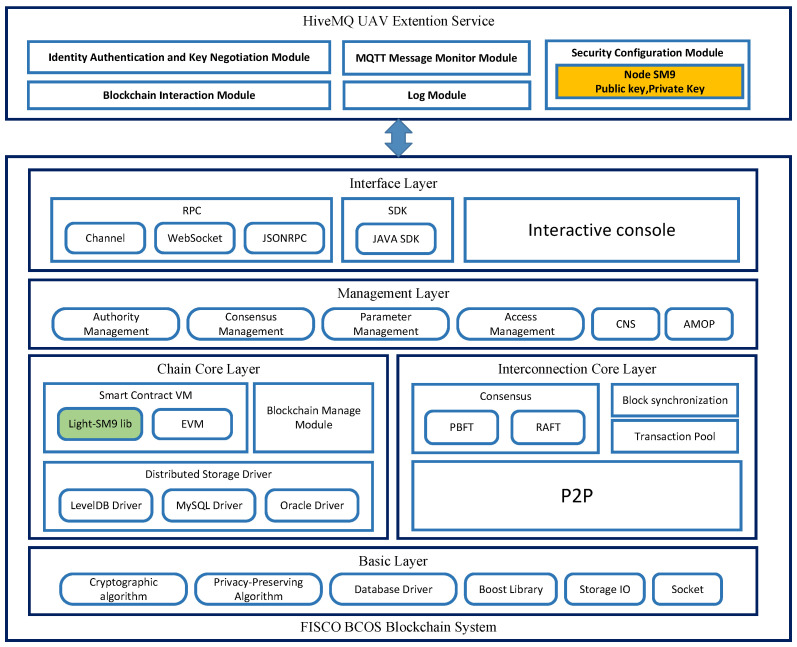
Blockchain System Node Architecture.

The architecture of the blockchain-enable drone identity authentication and secure communication system, as shown in Figure 5, utilizes the open-source consortium blockchain system FISCO BCOS as its underlying technology. The MQTT drone Extension service is built on the consortium blockchain system. The consortium blockchain system comprises the Basic Layer, Chain Core Layer, Interconnection Core Layer, Management Layer, and Interface Layer.

The blockchain network is composed of multiple permissioned nodes (four in our prototype) that collectively maintain the ledger through a consensus protocol (FISCO BCOS supports PBFT and Raft). Each blockchain node is hosted on a server in the ground control platform, and also runs a co-located MQTT broker instance as part of the integrated system (forming the HiveMQ cluster). This means the ground control platform is distributed across those nodes—there is no single centralized server handling all authentications. Instead, any broker/node can process an authentication request by invoking the blockchain smart contract, and the result is agreed upon via consensus. Importantly, FISCO BCOS requires each node to have proper identity credentials (certificates) to join the consortium, adding a layer of security: only authorized control-platform servers can participate in authentication processing. The drone devices do not run blockchain clients rather, they communicate with the nearest broker, and the broker interacts with the blockchain on their behalf.

Identity registration and multi-signature: Before drones are deployed, an administrator (or manufacturer) uses a KGC to generate each drone’s private key (using the SM9 IBC scheme, discussed later). The corresponding public parameters (identity ID and public key) are then registered on the blockchain. We implemented this as a blockchain transaction that adds the drone’s ID and public key to a registry contract on FISCO BCOS. To enhance trust, the registration transaction utilizes FISCO BCOS’s multi-signature mechanism. This means multiple authorities (e.g., two-out-of-three designated admin keys, or all four consortium nodes) must approve the addition of a new drone identity. Only if the required threshold of signatures is provided will the blockchain accept and record the new identity. This multi-signature approach prevents a single compromised authority from illegitimately adding drone identities, thus distributing the trust in the enrollment stage. Once confirmed, the drone’s identity record (ID → public key mapping) is stored on-chain in an immutable manner. All blockchain nodes have a consistent copy of this data, and drones themselves can later query or receive proof of their registration if needed (the scheme currently uses the broker to check on behalf of the drone). The use of a consortium blockchain also means that even if one or two node servers fail or go offline, the remaining nodes can continue to verify identities and preserve the integrity of the identity ledger.

In our proposed scheme, to improve the security of cellular networked UAV, we adopt the SM9 as the encryption algorithm. SM9 is a class of identity-based cryptographic algorithms based on bilinear pairing, and is incorporated into the international standard in 2021 (ISO/IEC 18033-5 AMENDMENT 1) [18].

Our scheme utilizes SM9 for both mutual authentication and key agreement between drones and the ground station (or between drones)—this means that when a drone and the ground platform authenticate each other, they also establish a shared session key in the process, with no additional Diffie-Hellman exchange needed. The appeal of SM9 in the UAV context is its low communication overhead (no digital certificates to transmit or verify) and the simplified key management (public keys are just identities, and private keys are issued by the KGC). However, a challenge is that SM9’s cryptographic operations, especially the bilinear pairings, are computationally heavy for resource-constrained devices. A naive implementation could be too slow on drones or introduce too much latency.

SM9 in our system: We address this by offloading the heaviest operation—the bilinear pairing verification—to the powerful servers (blockchain nodes) via the precompiled contract as described above. In the SM9 authentication, the drone must generate an authentication token (which typically involves computing a pairing or exponentiation with its private key and a random challenge). The ground station (HiveMQ plugin) then uses the drone’s public identity and the SM9 pairing function on the blockchain to verify that token. Concretely, SM9 defines that given an identity $ID_{C}$ and the KGC’s master public key, the drone’s public key can be derived, and the drone possesses a private signing key $d_{C}$ derived from the master secret. In an authentication exchange, the drone sends some value (often denoted $\phi_{C}$ and a signature $\sigma_{C}$ in literature) computed with $d_{C}$ and a random nonce. The verifier checks this via a pairing equation, e.g., verifying $e\left(\sigma_{C},P\right)=e\left(H\left(ID_{C},nonce\right),P_{pub}\right)$ or a similar construct, where $P_{pub}$ is the KGC’s master public key and *H* stands for hash operation. This pairing equation must hold true only if the drone’s token is valid. Thanks to the precompiled SM9 contract, the blockchain can evaluate this equation quickly. If valid, it essentially means the drone is authenticated (its identity is confirmed) and both parties can now compute a shared session key. In SM9 key agreement, typically each side contributes a nonce ($n_{C}$ for the drone, $n_{B}$ for the base station), and after the exchange both can derive the same session key using a combination of these nonces and static identity keys. Our implementation follows this approach: a secure hash function is applied to the keying material (including both parties’ public keys and nonces) to derive the session key. This key is then used to secure subsequent communications (e.g., encrypting control commands or telemetry using symmetric cipher).

To implement the proposed scheme, the system integrates the Light-SM9 algorithm’s C language library into the Chain Core Layer, providing interfaces for both the MQTT UAV Extension service and the EVM (Ethereum Virtual Machine), significantly enhancing the efficiency of authentication and key negotiation processes.

The MQTT UAV Extension service encompasses several modules: the Identity Authentication and Key Negotiation Module, Blockchain Interaction Module, MQTT Message Monitor Module, Security Configuration Module, and Log Module. The Identity Authentication and Key Negotiation Module handles the identity authentication and key negotiation for UAVs. The unique NodeID information for blockchain nodes is generated by combining the MAC address information of the node’s computer with the CPU ID information. The Security Configuration Module ensures a secure and reliable environment for storing private keys generated by the KGC based on NodeID.

### 5.2. Precompiled Contract for Bilinear Pairing Algorithm

Due to the complexity and heavy computational demands of the SM9 bilinear pairing verification algorithm, which involves intricate elliptic curve cryptography, directly implementing this algorithm within smart contracts would result in significant computational costs and low execution efficiency. To address this issue, the bilinear pairing verification algorithm implemented through a combination of C language and assembly programming is embedded as a precompiled contract into the smart contract virtual machine (EVM), tremendously improving the efficiency of identity authentication smart contracts executed by the EVM. The use of the bilinear pairing precompiled contract also ensures accuracy and consistency in identity authentication computations across the blockchain system. By integrating complex computations into the underlying layer of the EVM, the precompiled contract provides a more reliable and secure computation method, reducing the risks of potential errors and security vulnerabilities. This approach not only enhances operational efficiency but also strengthens the security and stability of the blockchain system.

**Figure 6:**
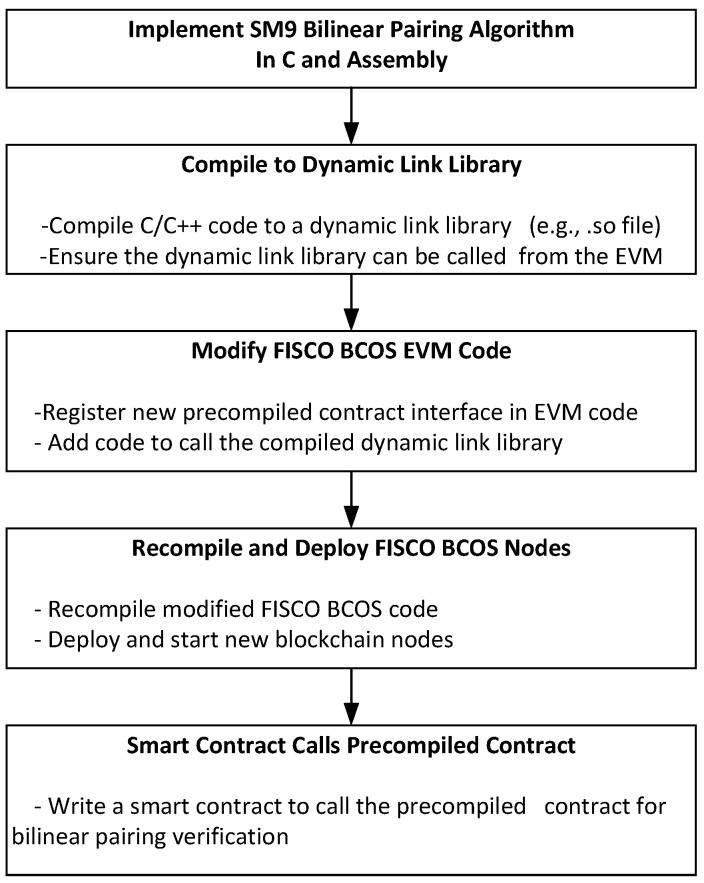
Bilinear Pairing Precompiled Contract Implementation Steps.

As shown in Figure 6, the steps to implement the bilinear pairing precompiled contract are outlined as follows. Firstly, the bilinear pairing operation is implemented using C language and assembly to ensure high computational performance. Secondly, this implementation is compiled into a dynamic link library (e.g., a .so file). Then, a new precompiled contract interface (address(0×12)) is registered within the FISCO BCOS EVM code, enabling it to invoke the bilinear pairing operation from the dynamic link library. Secondly, the FISCO BCOS node, including the precompiled contract, is recompiled and deployed to ensure it can correctly load and call the new precompiled contract. Lastly, smart contracts are written to call the precompiled contract for bilinear pairing verification, facilitating efficient identity authentication or other cryptographic operations. Through these steps, the bilinear pairing operation can be efficiently and securely implemented within the EVM, improving the execution efficiency and security of the blockchain system. The final bilinear precompiled contract, as depicted, allows other smart contracts to invoke the verify function within the LightSM9Verifier contract.

    contract LightSM9Verifier {

       function verify(bytes memory H1, bytes memory H2,

                             bytes memory Q1, bytes memory Q2) public returns (bool) {

          bytes memory input = abi.encodePacked(H1, H2, Q1, Q2);

          (bool success, bytes memory result) = address(0×12).call(input);

          require(success, “Precompiled contract call failed”);

          bool valid = abi.decode(result, (bool));

          return valid;

          }

    }

### 5.3. Integration of HiveMQ and FISCO BCOS Blockchain

HiveMQ, through its plugin integration with the FISCO BCOS blockchain system, effectively implements functions such as UAV registration, identity authentication and key agreement, and key updating. In this framework, the HiveMQ plugin utilizes the Java API of FISCO BCOS to invoke smart contracts, ensuring the security and efficien-cy of the entire process.

Firstly, in the drone registration stage, the administrator, operating at the KGC, procures the public and private key pair pertinent to each drone. Subsequently, the drone’s identification and public key are inscribed onto the blockchain via the HiveMQ plugin, thereby safeguarding the integrity of the registration protocol.

Secondly, in the identity authentication and key agreement stage, UAVs establish a connection to the HiveMQ Broker through the MQTT protocol. Harnessing the capabilities of the HiveMQ plugin, they dispatch identity authentication requests, ensuring a secure and authenticated exchange.

Lastly, in the UAV key update stage, administrators employ the HiveMQ plugin to exploit FISCO BCOS’s Java API, facilitating the submission of updated public keys for key renewal. This process underpins the relentless management of cryptographic credentials, upholding the security framework’s integrity.

### 5.4. HiveMQ Broker and Redis Cluster Configuration

To ensure high availability and scalability of the system, a HiveMQ Broker cluster is also configured. Firstly, HiveMQ Broker instances are deployed on all blockchain node servers to form a cluster architecture. Each node runs the same HiveMQ plugin to ensure consistent identity authentication and key negotiation functionality. Secondly, cluster communication is configured to enable nodes to communicate and synchronize their states with each other. This communication is achieved through the TCP/IP protocol, ensuring high availability and load balancing of the cluster. Further-more, Redis instances are deployed across multiple nodes to construct a highly available Redis cluster for storing session keys. The Redis cluster provides data persistence and efficient access, ensuring the secure storage and rapid retrieval of session keys, thereby enhancing the overall performance and reliability of the system.

## 6. Security Analysis

In the real world, cyber attacks on drones primarily aim to steal sensitive data, hijack control for malicious purposes like espionage or physical attacks, or disrupt their operations through jamming. These threats target commercial, and personal drones for motives ranging from national security breaches to privacy invasion and financial gain.

In wireless network security, the Dolev-Yao threat model is widely adopted, characterizing attackers with capabilities such as eavesdropping, intercepting, tampering, and fabricating messages, as well as impersonating legitimate network identities. However, this model does not fully address the security dynamics of drone networks. UAVs must register at a ground control station via a secure channel, which significantly mitigates the risk of information interception during registration. Furthermore, the use of consortium blockchain in this study enhances security by requiring certificate-based authentication for node participation. Given these protections, this paper argues that the Dolev-Yao assumption of attackers possessing legitimate identities is excessively strong and misaligned with the realities of UAV networks.

Accordingly, this paper adapts the Dolev-Yao model by removing the assumption that attackers hold legitimate network identities. This revision better reflects UAV network security needs and avoids overly conservative defenses that could unnecessarily drain computational and real-time processing resources. Focusing on relevant attack vectors, UAV networks face threats such as impersonation, identity forgery, eavesdropping, and man-in-the-middle attacks. It will be demonstrated that the proposed algorithms and system effectively counter these four types of attacks.

### 6.1. Identity Forgery Attack

This solution possesses the capability of bidirectional identity authentication, rendering it impossible for attackers to utilize existing or received information to falsely impersonate or forge legitimate identity authentication credentials. In the authentication key agreement protocol, the authentication of an entity’s identity is equivalent to establishing the same session key, thereby gaining the status of a legitimate participant in the drone network and potentially stealing data. The following demonstrates that attackers cannot successfully execute counterfeit or forgery attacks in any part of this scheme to obtain the same session key as legitimate drone nodes or ground control stations in the drone network.

1. Attack Scenario 1: During the authentication key agreement phase between drone nodes and blockchain network nodes, attackers attempt to pass identity authentication through forgery or impersonation, aiming to generate the same session key as drone nodes or ground control stations.
  Defense Analysis: In the authentication phase, the input of the session key generation function includes secret information stored in the entity. Taking a ground control station and server as an example, the session key is $sk_{B}=\mathrm{Hash}(Key_{B-pub}||Key_{C-pub}\left|\right|\phi_{B}\left|\right|\phi_{C}\left||X\right)$. Due to the one-way nature of hash functions, attackers must input identical elements to calculate the session key. Among these elements, X contains secret information from both the consortium blockchain node B and drone node C. As attackers lack the ability to guess the securely stored secret information of entities, they cannot obtain the correct session key through forgery or impersonation. Therefore, Attack Scenario 1 is invalid.
2. Attack Scenario 2: During the authentication key agreement phase between drone nodes, attackers attempt to pass identity authentication through forgery or falsified messages, aiming to generate the same session key as a drone node.
  Defense Analysis: The bidirectional identity authentication key agreement process between drone nodes involves message exchange through both encrypted and public channels. Messages transmitted through encrypted channels cannot be forged by attackers due to their inability to decrypt SM4 symmetric encryption algorithm ciphertext. The only message transmitted through the public channel is a message sent from drone node C2 to drone node C1, which is $Key_{C2-pub},\phi_{C2},\sigma_{C2}$. As proven in Attack Scenario 1, based on the computational difficulty of bilinear pairings, attackers cannot construct messages that can pass the bilinear pairing verification of drone node C1. Therefore, both secure and public channel messages are unforgeable, rendering Attack Scenario 2 nonexistent.
3. Attack Scenario 3: During the key update phase of drone nodes, attackers attempt to replace the key pair information sent by the ground control station through forgery or impersonation.
  Defense Analysis: The key pair update information sent by the ground control station during the key update phase is transmitted through encrypted channels. Since attackers cannot decrypt or generate ciphertext, they cannot replace the intended message. Therefore, Attack Scenario 3 is invalid.

### 6.2. Eavesdropping Attack

According to the definitions in the threat model, attackers in the drone network can obtain any message in the network, but the types of attacks they can execute after obtaining the messages are limited to two: directly decrypting the ciphertext to obtain the plaintext and using the obtained messages to carry out other attacks.

1. Attack Scenario 4: Attackers crack and obtain the plaintext of encrypted messages based on any eavesdropped message in the drone network.
  Defense Analysis: Under the assumptions of the proven threat model, attackers cannot decrypt and obtain the plaintext from the ciphertext of a sufficiently secure symmetric encryption algorithm. Therefore, Attack Scenario 4 is invalid.
2. Attack Scenario 5: Attackers use any eavesdropped message in the drone net-work to carry out other types of attacks.
  Defense Analysis: This attack scenario is invalid. The proof process is detailed in the defensive proofs against other types of attacks.

### 6.3. Man-in-the-Middle Attack

Attackers attempt to forward messages between two normally communicating entities by posing as a man-in-the-middle, disguising themselves as the correct communication partner to both sides of the communication, in order to achieve the attack goal of obtaining critical information or legitimate identities. The key update phase is a one-way communication from the ground control station to the drone, with no information exchange, and thus there is no risk of a man-in-the-middle attack. Therefore, we only need to discuss the defense capabilities against man-in-the-middle attacks in the authentication key agreement phase.

1. Attack Scenario 6: In the authentication key agreement phase, the attacker launches a man-in-the-middle attack to obtain the same session key as a certain entity, thereby gaining a legitimate identity.
  Defense Analysis: In the authentication key agreement phase, attackers may at-tempt to intercept and forward messages to disguise themselves as legitimate entities. Taking the interaction between drone node C and ground control station B as an ex-ample, an attacker may intercept and attempt to forward messages from node C to disguise as node C. However, the attacker cannot construct a valid forged message to pass the bilinear pair verification due to the unknown critical unpublished curve generator and the node’s authentication public key, as well as the lack of secret information required to generate a valid session key. Therefore, the attacker cannot generate the same session key as the drone or ground control station during the authentication key agreement process, making the attack scenario invalid.
2. Attack Scenario 7: The attacker attempts to launch a man-in-the-middle attack during the authentication key agreement protocol execution between drone node C1 and drone node C2.
  Defense Analysis: The attacker may intercept messages in the secure channel or the public channel and attempt to replace them for key exchange. However, information in the secure channel is protected by SM4 symmetric encryption, and the at-tacker cannot generate the corresponding ciphertext for tampering. If public channel information is replaced, based on the computational difficulty of bilinear pairs, the at-tacker cannot generate a session key that matches the legitimate drone nodes. There-fore, a man-in-the-middle attack between drone nodes is also unlikely to succeed.

## 7. Experimental Evaluation

To validate the performance, scalability, robustness, and resource efficiency of the proposed Blockchain-enabled Identity-Based Cryptography (SM9) Authentication System for Cellular-Connected Drones, and to compare it against centralized PKI and centralized IBC (SM9/KGC) baselines, we designed and executed 5 categories of experiments. These experiments cover key dimensions including throughput and latency, network impairments, concurrent access, key update overhead, and resource consumption. All experiments were conducted with the computational upper bound of a single-machine SM9 implementation at approximately 220 operations per second (ops/s). The blockchain system utilized a multi-group parallel architecture comprising 20 nodes partitioned into 5 groups of 4 nodes each. The system-level confirmed throughput was observed to be stable at approximately 1000–1100 TPS, with a typical value of 1045 TPS within a 200s observation window.

The blockchain platform is FISCO BCOS, a permissioned chain employing the PBFT consensus algorithm. The SM9 bilinear pairing verification is integrated into the EVM as a precompiled contract. The access layer is implemented as an MQTT Broker cluster, which invokes on-chain smart contracts via a plugin, complemented by a Redis cluster for persistent session key storage. The server hardware consists of Intel® Xeon® E5–2630 v3 CPUs, 64 GB RAM, and 1 TB SSDs. The software stack includes FISCO BCOS 2.8, HiveMQ 2024.1, Redis 7.2.5, and JDK8. Unless otherwise specified, all throughput and success rate metrics were measured over a 200 s window, and TPS refers to the system-level confirmed throughput.

### 7.1. Scalability of the Multi-Group Parallel Architecture for Distributed UAV Identity Authentication

This experiment was designed to validate the effectiveness of the multi-group parallel architecture in enhancing the overall system throughput. We deployed a total of 20 nodes on the same physical cluster, partitioned into 5 independent 4 node PBFT consensus groups. Each group maintains a separate ledger and executes smart contracts independently. By incrementally increasing the number of active parallel groups from one to five, we measured and compared the aggregated transaction throughput (TPS) to assess the system’s horizontal scalability.

Compared to Hyperledger Fabric, FISCO BCOS natively supports a multi-group parallel mechanism, where each group, with its independent ledger and PBFT committee, can operate in isolation and parallel on the same physical cluster. In our UAV identity authentication use case, a single on-chain transaction requires only one SM9 signature verification (k=1), and the system-level confirmed throughput of a single 4 node group was measured at approximately 220 TPS within a 200s observation window. Theoretically, as long as new groups are formed with non-overlapping node committees and are allocated sufficient computational, storage, and network resources, the total system throughput can achieve near-linear growth according to the ideal model of “Number of Groups × 220 TPS”.

**Figure 7:**
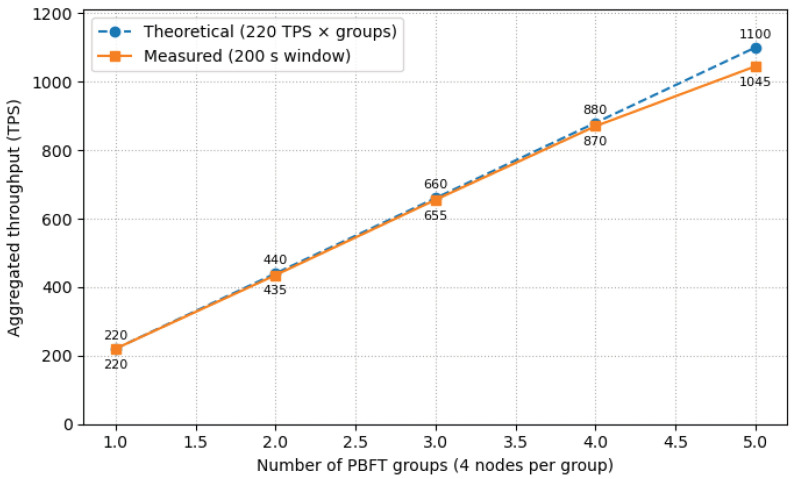
Scalability of Multi-Group Parallel Architecture for UAV Distributed Identity Authentication.

As illustrated in the Figure 7 below, within our 20-node deployment, we constructed 5 independent 4-node groups. The experimental results show that over a 200s observation window, the aggregated throughput reached approximately 1000– 1100 TPS, with a typical value of 1045 TPS. This measured performance aligns closely with the theoretical expectation of 1100 TPS (5 groups × 220 TPS/group), strongly validating the high scalability of the FISCO BCOS multi-group parallel architecture.

The experimental results clearly demonstrate that the multi-group parallel architecture provides a near-linear improvement in system-level throughput. The performance baseline for a single 4 node group is approximately 220 TPS, and with five groups operating in parallel, the aggregated throughput stabilizes at around 1045 TPS, which approaches the theoretical linear growth expectation.

This architecture holds decisive engineering significance for large-scale cellular-connected drone network applications. In practical operations, a massive number of drones may issue concurrent authentication requests, where the processing capacity of a single group could easily become a bottleneck, leading to queuing delays and reduced real-time performance. By partitioning groups based on geographical airspace or mission type, “partitioned authentication with network-wide parallelism” can be achieved. This design not only effectively enhances system capacity through horizontal scaling but also improves fault tolerance and security via inter-group isolation, preventing latency jitter caused by single-point congestion and laying a solid technical foundation for the trusted access and real-time coordination of tens of thousands of future UAVs.

**Figure 8:**
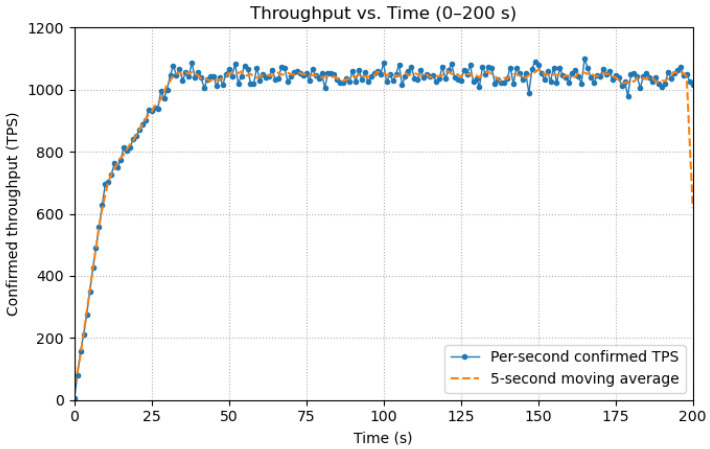
Throughput vs. time (0–200 s) for the proposed system (5 disjoint 4 node PBFT groups, SM9 precompiled verification, *k* = 1).

The curve in Figure 8 shows a short warm up (0–30 s) during which sessions, tx pools and consensus pipelines are primed, followed by a steady state at 1045TPS. At *t* = 0–10 s, client connections (MQTT sessions) and initial transaction queues are established. At *t* = 10–30 s, the PBFT pipeline, block sealing cadence, and per-group batching reach a stable rhythm across the five groups. From *t* = 30 s onward, the system operates at a steady state around 1045 TPS, with minor oscillations due to 1s block sealing and routine retransmissions. This profile confirms that our reported capacity numbers are not momentary spikes but sustained plateaus once the system is warmed up.

### 7.2. Authentication Success Rate Under Adverse Network Conditions

To evaluate the scheme’s reliability in adverse network environments, this experiment simulated two typical network impairments between the terminal and the blockchain network: increased Round-Trip Time (RTT) and packet loss. We configured various packet loss rates (0%, 5%, 10%, 15%, 20%) and two latency settings (low latency at 10 ms, high latency at 200 ms), measuring the authentication success rate under a 1-s timeout threshold. The experiment was conducted on the 5-group parallel architecture with a maximum system throughput of approximately 1045 TPS.

The authentication success rate is defined as the proportion of requests that successfully complete the authentication handshake within the specified timeout period. Increased network latency prolongs message round-trip times, while a higher packet loss rate increases the probability of message retransmissions or timeout failures; both factors can negatively impact the success rate. 

**Figure 9:**
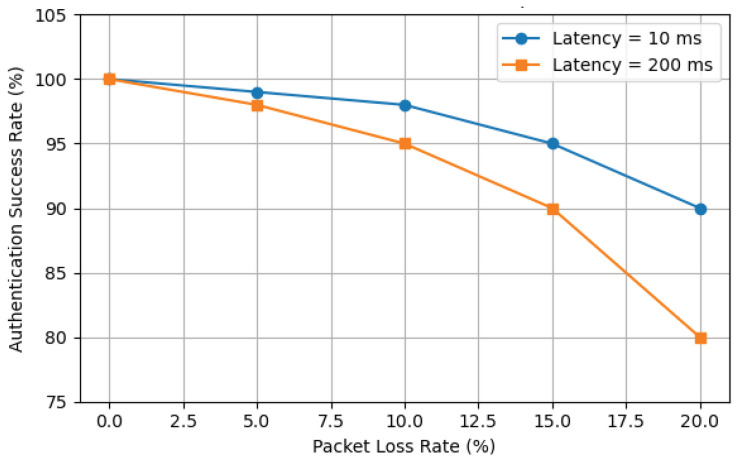
Success Rate under Network Impairments.

The experimental results are shown in the Figure 9. These results quantify the cumulative impact of network impairments on the authentication success rate. A high packet loss rate is the primary cause of the decline in success rate, an effect that is exacerbated by higher network latency. Nevertheless, the results also demonstrate the robustness of the proposed scheme: under moderate network anomaly conditions (e.g., 15% packet loss), the system can still maintain an authentication success rate of no less than 90%. A significant drop in success rate only occurs in very harsh network environments. This validates that the proposed distributed authentication mechanism maintains a high degree of reliability even over unstable communication links.

### 7.3. Authentication Latency Distribution Under High-Concurrency Multi-Terminal Access

This experiment was designed to evaluate the system’s quality of service under high concurrent loads, with a particular focus on the distribution characteristics of authentication latency. We simulated a scenario where 1000 UAV terminals initiate authentication requests simultaneously. These requests were distributed among 5 parallel consensus groups, with the assumption that each group could stably package and confirm one block per second. The core metric was the end-to-end completion time for each request, from initiation to final confirmation, presented as a Cumulative Distribution Function (CDF).

The system’s theoretical maximum throughput is approximately 1045 TPS, meaning the load from 1000 concurrent requests is close to but does not exceed the system’s capacity limit. Under these conditions, requests are distributed roughly evenly among the 5 groups. We anticipated that the vast majority of requests would be processed and confirmed within the first block period (approx. 1 s), with only a small fraction assigned to momentarily busy groups potentially being deferred to the second block period.

**Figure 10:**
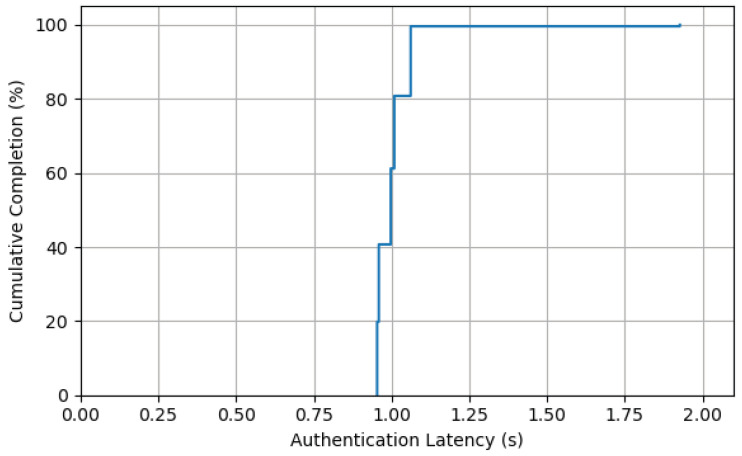
Latency Distribution (1000 Concurrent Authentications).

The CDF curve shown in the Figure 10 accurately reflects this model. The data indicate that approximately 80% of requests completed authentication within 1.0 s. The 95th percentile (P95) latency was approximately 1.07 s, demonstrating a highly consistent and low-latency experience for the vast majority of users. These results clearly demonstrate that the proposed scheme can provide low and stable authentication latency even when operating near full capacity. This experiment strongly proves the system’s efficiency and service stability under large-scale concurrent scenarios, showcasing its capability to handle bursty, high-density access requests in practical applications.

### 7.4. Impact of Dynamic Key Update Frequency on Authentication Performance

**Figure 11:**
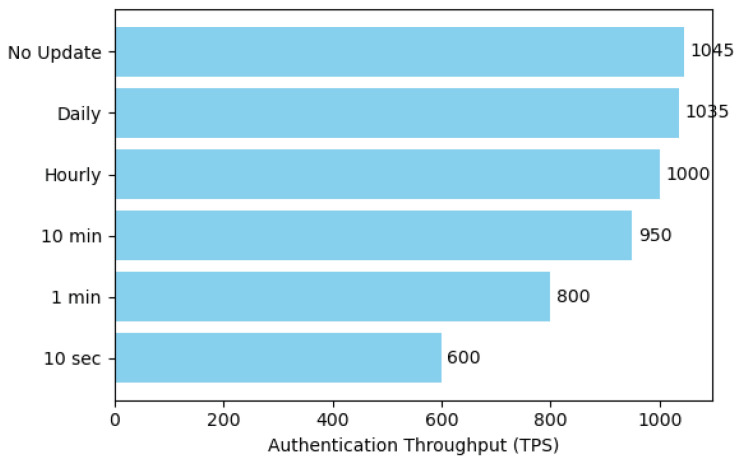
Throughput vs. Key Update Frequency.

This experiment aimed to quantify the impact of the dynamic key update security mechanism on the system’s authentication performance. As shown in Figure 11, we configured several update frequencies, ranging from no updates to high-frequency updates, including “No Update,” “Daily,” “Hourly,” “10 min,” “1 min,” and “10 sec.” For each frequency setting, we measured the maximum sustained authentication throughput (TPS) the system could maintain. We hypothesized that a higher update frequency would consume more computational and on-chain consensus resources, thereby increasingly impacting the normal authentication processing capacity.

Dynamic key updates enhance forward secrecy by periodically changing keys, but the processes of generation, distribution, and synchronization inevitably consume system resources. The horizontal bar chart clearly illustrates the trend of authentication throughput decreasing as the key update frequency increases.

Under the baseline condition of no key updates, the system achieved its peak throughput of approximately 1045 TPS. A daily update frequency (1035 TPS) had a negligible impact on performance. When the frequency was increased to hourly, the throughput slightly decreased to 1000 TPS, an acceptable performance loss. However, as the update frequency was further accelerated to every 10 min (950 TPS) and every 1 min (800 TPS), the performance degradation became significant. Notably, with updates every minute, the throughput loss exceeded 20%. In the extreme high-frequency scenario of updating every 10 s, the system throughput dropped sharply to about 600 TPS, which is only about 57% of the peak performance, as a substantial portion of system resources was dedicated to processing key update transactions.

The results quantify the trade-off between security policy and system performance. Low-frequency key updates (e.g., daily or hourly) have a minimal effect on the system’s peak throughput. However, when the update frequency exceeds the per-minute level, the associated performance overhead becomes significant. Therefore, in practical engineering, an update frequency of every hour or every 10 min is recommended as a balanced point, which can meet the security requirements of most scenarios without imposing an excessive impact on the system’s authentication performance.

### 7.5. System Resource Consumption Comparison

**Figure 12:**
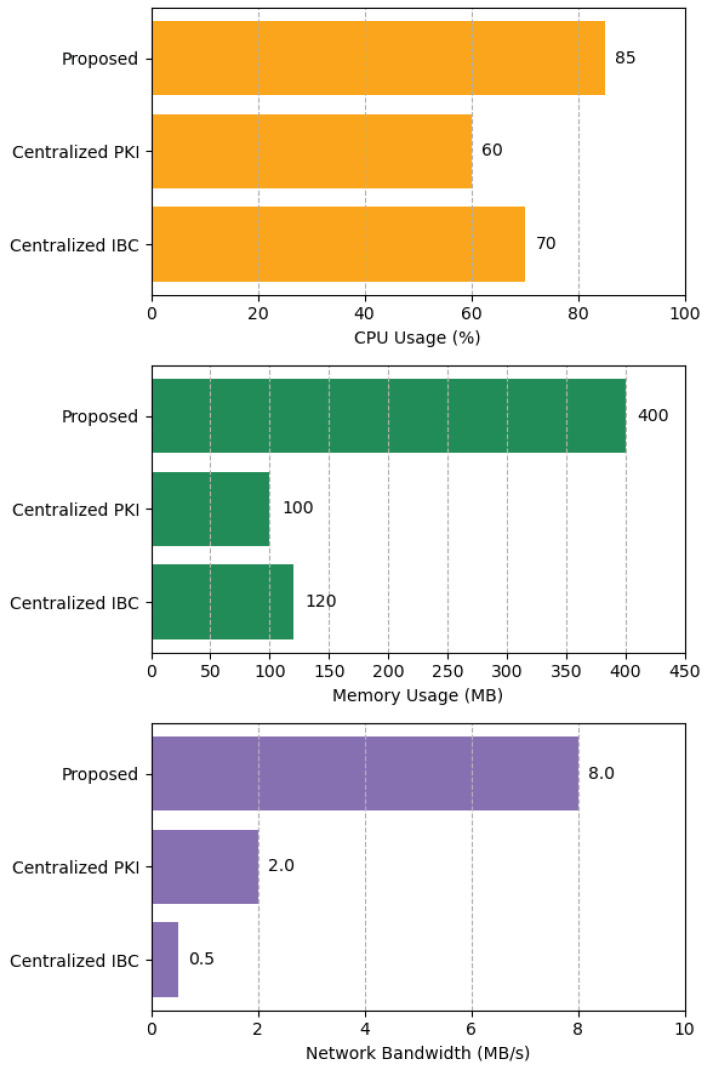
CPU Usage, Memory Usage and Network bandwidth.

This experiment aimed to quantitatively assess the differences in resource consumption between our proposed distributed scheme and traditional centralized approaches. Under a steady-state high load of approximately 1000 TPS, we compared three architectures. (1) The proposed scheme (multi-group blockchain + SM9 precompile). (2) A centralized PKI scheme (certificate-based authentication). (3) A centralized IBC scheme (KGC based on SM9). The core comparison metrics included CPU utilization, memory usage, and network bandwidth. To achieve distributed consensus, multi-copy ledgering, and decentralized trust, a blockchain architecture is expected to incur higher resource overhead than centralized architectures. The experimental results, as shown in Figure 12, confirmed this expectation, with our proposed scheme consuming significantly more resources across all three metrics compared to the two centralized schemes.

CPU Utilization: Due to the need to execute multi-copy SM9 operations and consensus algorithms, the average CPU utilization of our proposed scheme reached approximately 85%. In contrast, the CPU usage of the centralized PKI (primarily traditional signature verification) and centralized IBC (no consensus overhead) was about 60% and 70%, respectively.

Memory Usage: A single blockchain node in our scheme required about 400 MB of memory to maintain the ledger, consensus state, and execution stack. The centralized PKI server and IBC’s KGC, having more streamlined architectures, consumed only about 100 MB and 120 MB of memory, respectively.

Network Bandwidth: The resource overhead difference was most prominent in network bandwidth. Because transactions and blocks must be broadcast among consensus nodes, the internal network traffic was as high as 8.0 MB/s. In contrast, communication in the centralized PKI and IBC schemes occurs mainly between the terminal and the server, resulting in bandwidth consumption of only 2.0 MB/s and 0.5 MB/s, respectively.

These results in Figure 12 illustrates that our distributed blockchain architecture incurs higher computational, storage, and network overhead compared to a centralized baseline. This overhead is the direct result of the system’s strengthened security and trust mechanisms—for instance, performing consensus and replicating data across nodes to prevent tampering. Such costs are justified by the corresponding gains in security and reliability: the scheme deliberately trades additional resources for robust, trust-free operations that centralized schemes cannot attain. Therefore, the increased resource usage is an inherent investment in security, rather than inefficiency. In scenarios with high requirements for decentralization, security, and scalability, the resource investment of our proposed scheme is both reasonable and necessary. Conversely, in resource-constrained environments or scenarios with a higher tolerance for single-point-of-failure risks, traditional centralized schemes are more cost-effective.

This section provided a comprehensive evaluation of the proposed large-scale UAV authentication scheme, which is based on blockchain and the SM9 algorithm, through a series of systematic experiments. The differentiating contributions of this work, as demonstrated in the experiments, are primarily manifested in the following areas.

Optimization of On-Chain Cryptographic Primitives: We innovatively integrated the computationally intensive SM9 signature verification operation into the blockchain’s execution layer as a precompiled contract, significantly reducing the on-chain transaction processing overhead and laying the foundation for high-throughput authentication.

Decoupled and Parallel Architecture Design: a decoupled access model of “MQTT with Blockchain” effectively isolates IoT device connectivity pressure from blockchain consensus overhead. Simultaneously, through a multi-group parallel consensus mechanism, near-linear scalability of the system’s processing capacity is achieved.

In conclusion, the experimental results demonstrate that by organically combining innovative designs such as on-chain SM9 execution, decoupled access, and multi-group parallelism, our proposed scheme successfully elevates the system-level capacity of the authentication service to the thousand-TPS level while ensuring the security and consistency of a distributed system. This level of performance and governance capability is significantly superior to that of traditional centralized PKI and centralized IoT blockchain platforms, offering a viable technical path to address the challenges of identity authentication and trust establishment in large-scale cellular-connected drone scenarios.

## 8. Conclusions

In summary, this paper presents a novel distributed system for drone identity authentication and key agreement, built upon SM9, the high-performance consortium blockchain and MQTT. The system achieves efficient and secure identity authentication and key agreement, ensuring data immutability and traceability through blockchain technology. The innovations of this paper lie in combining the SM9 algorithm and blockchain technology to achieve high-security identity authentication, utilizing the MQTT broker for efficient key management, and implementing dynamic key updates through multisignature and smart contracts. Experimental results demonstrate that the system operates stably under high-load environments, providing an efficient and secure communication solution for drone swarms. Despite the promising results, this study has certain limitations. The proposed system introduces additional complexity by integrating multiple components (blockchain, MQTT cluster), which may increase deployment and maintenance overhead. Future research will focus on validating the system’s performance in large-scale real-world drone deployments.

## Figures and Tables

**Table 1:** Characteristics of related works.

Schemes	Type	Core Technology	Degree of Centralization	Advantages	Limitations
Ozmen and Yavuz [15]	PKI	Public key certificate, CA	Centralization	Mature and Standardized	Complex certificate management and the risk of a single point of failure
Das et al. [10]	PKI	t-collusion resistant, t-degree symmetric bivariate polynomial	Centralization	High Anonymity and Untraceability	Risk of single point of failure, and excessive communication costs
Wani et al. [20]	IBC	Identity public key, bilinear pairing	Centralization	Certificate-free, simplifying operations	Key escrow problem and KGC single point of failure
Tan et al. [22]	Blockchain	Blockchain, smart contract	Decentralization	Distributed and immutable ledger	The challenges of on-chain interactions: high cost and significant latency
Du et al. [27]	Blockchain	Blockchain	Decentralization	Reduced burden, enhanced security	Dependency on fog nodes, restricted experimental scale
Shahidinejad et al. [28]	Blockchain	Blockchain, Chebyshev chaotic map	Decentralization	Cost-effective and a high level of anonymity	High dependence on the GSS network
This paper	Blockchain + MQTT	Blockchain, smart contract, MQTT	Decentralization	Decentralized, tamper-resistant, and scalable	Reliance on the coverage of the cellular network

## Data Availability

Correspondence and requests for materials should be addressed to Zeyuan Li.
